# Deletion of IP6K1 in mice accelerates tumor growth by dysregulating the tumor-immune microenvironment

**DOI:** 10.1080/19768354.2022.2029560

**Published:** 2022-01-31

**Authors:** Haein Lee, Seung Ju Park, Sehoon Hong, Seol-Wa Lim, Seyun Kim

**Affiliations:** a Department of Biological Sciences, Korea Advanced Institute of Science and Technology (KAIST), Daejeon, Republic of Korea; b KAIST Institute for the BioCentury, KAIST, Daejeon, Republic of Korea

**Keywords:** inositol hexakisphosphate kinase 1, tumor growth, tumor microenvironment, immunity

## Abstract

A family of inositol hexakisphosphate kinases (IP6Ks) catalyzes the production of inositol pyrophosphate IP_7_ (5-diphosphoinositolpentakisphosphate) which is known to modulate various biological events such as cell growth. While targeting IP6K1 in various cancer cells has been well reported to control cancer cell motility and invasiveness, the role of host IP6K1 in tumor progression remains unknown. By using a syngeneic MC38 murine mouse colon carcinoma model, here we examined how host IP6K1 in the tumor microenvironment influences tumor growth. In IP6K1 knockout (KO) mice, the growth of MC38 tumor cells was markedly accelerated and host survival was significantly shortened compared with wild-type (WT). Our flow cytometric analysis revealed that tumors grown in IP6K1 KO mice had lower immune suppressive myeloid cells and M1 polarized macrophages. Notably, infiltration of both antigen-presenting dendritic cells and CD8^+^ cytotoxic T lymphocytes into the tumor tissues was remarkably abrogated in IP6K1 KO condition. These studies suggest that enhanced tumor growth in IP6K1 KO mice resulted from reduced anti-tumor immunity due to disturbed immune cell actions in the tumor microenvironment. In conclusion, we demonstrate that host IP6K1 acts as a tumor suppressor, most likely by fine-tuning diverse tumor-immune cell interactions, which might have implications for improving the host response against cancer progression.

## Introduction

Inositol pyrophosphates are a class of high-energy signaling molecules characterized by the presence of pyrophosphate and monophosphate substitutions on the inositol ring (Chakraborty et al. 2011; Chakraborty 2018; Park et al. 2018). They participate in many cellular functions by binding specific proteins or by transferring their β phosphate to pre-phosphorylated serine residues to bring about serine pyrophosphorylation (S. Lee et al. 2020). The most abundant inositol pyrophosphate in mammals, 5-diphosphoinositol pentakisphosphate (5-PP-IP_5_ or 5-IP_7_) is synthesized from inositol hexakisphosphate (IP_6_) by inositol hexakisphosphate kinases (IP6Ks) (Saiardi et al. 1999; Saiardi et al. 2001; Chakraborty et al. 2011). IP6Ks are found in all eukaryotes, from lower organisms such as yeast to mammals possessing three IP6 K isoforms, IP6K1, 2, and 3. Pleiotropic signaling actions of IP6K1 include promotion of insulin release in pancreatic β cells (Bhandari et al. 2008; Landrier et al. 2019), maintenance of genome integrity (Rao et al. 2014), platelet coagulation (Ghosh et al. 2013), Akt signaling (Chakraborty et al. 2010), as well as presynaptic vesicle cycling (Lee et al. 2017; Park et al. 2020).

Recent studies revealed key actions of IP6K1 in the control of tumorigenesis and cancer progression. When IP6K1 is depleted in mouse embryonic fibroblasts and cancer cells, they undergo cellular changes associated with decreased cell migration and dysregulated focal adhesion kinase (FAK) activity (Jadav et al. 2016; Fu et al. 2017). IP6K1-depleted HCT116 colorectal cancer cell xenografts showed reduced invasion in immunocompromised mice when compared with wild-type mice (Xu et al. 2020). Moreover, IP6K1 knockout (KO) in mice protects against 4-nitroquinoline-1-oxide (4-NQO)–induced carcinogenesis and diminishes the progression of invasive carcinoma (Jadav et al. 2016), suggesting IP6K1’s pro-tumorigenic action in cancer cells. However, regarding tumor formation and progression, the role of host IP6K1 in this pathology remains poorly understood. Given that that complex cellular interactions in the tumor microenvironment have been shown to play a major role in tumor growth and progression, we hypothesized that IP6 K in the tumor microenvironment could contribute to a host protective response against cancer.

In the present study, we investigated whether host IP6K1 KO could control tumor growth using a syngeneic MC38 model which is a murine colon adenocarcinoma tumor model on the C57BL/6 background. Using the global IP6K1 KO mice, we show that the absence of the host IP6K1 results in significantly shortened survival compared to normal mice due to more rapidly growing tumors. By flow cytometric analysis we showed that tumors in IP6K1 KO mice contain high levels of CD11b^+^Gr1 ^+ ^IL10^+^ myeloid cells (e.g., neutrophils, monocytes) and lower infiltration of M1-polarized tumor-associated macrophage (TAM), dendritic cells, and CD8^+^ killer T cells into tumors. Our data indicate that host IP6K1 acts as a tumor suppressor to regulate anti-tumor immune activities and control the recruitment and activation of cytotoxic T cells to the primary tumor through reprogramming tumor-immune control, and loss of this pathway increases tumor progression.

## Materials and methods

### Ethics statement

All mice were bred and housed under specific pathogen-free, temperature- and humidity-controlled conditions in a 12-hour light–dark cycle at KAIST. They received a standard laboratory chow diet and water ad libitum. All experiments involving animals were conducted according to the ethical policies and procedures approved by the Committee for Animal Care at KAIST.

### Mouse tumorigenesis and survival

For the tumor growth experiments, 6–7 weeks old male and female mice were inoculated MC38 colorectal cancer cells subcutaneously at both flanks of each mouse (2.5 × 10^5^ cells/side). After 7 days passed from tumor injection, mice's body weight and tumor size were measured once every 2 to 3 days. Tumor volume was evaluated according to the general formula 0.5 × (width)^2^ × (length) using a caliper, and Student's *t-test* was used to determine p-values. For mouse survival experiments, 12–14 weeks old male mice were subjected to inject MC38 cells (5.0 × 10^5^ cells/side) subcutaneously, and assessed survival rate and body weight daily. Mice were immediately euthanized when tumor volume exceeded 1000 mm^3^.

### Flow cytometry

Isolation of immune cells from tumor tissues was performed as described previously (Yang et al. 2021). Briefly, cells from tumor tissues were isolated and stained with anti-mouse CD45.2, anti-mouse CD11b, anti-mouse F4/80, anti-mouse CD80, anti-mouse CD206, anti-mouse CD11c, anti-mouse Gr-1, anti-mouse CD3, anti-mouse CD8, anti-mouse MHCII, anti-mouse IL-10, anti-mouse IFN-γ antibodies. Live cells were counter-stained with Fixable Viability Dye (e-bioscience) diluted 1:50 to 1:200 in fluorescence-activated cell sorting (FACS) buffer (DPBS containing 1% BSA and 0.5% sodium azide) followed by manufacture’s instruction. Stained cells were washed twice with 2 mL of FACS buffer. Flow cytometry data were acquired using a FACSDiva flow cytometer (BD Biosciences) and analyzed using the FlowJo software. For assessing DC development of IP6K1 KO mice, cells were isolated from thymus, bone marrow, spleen, and peripheral (mesenteric and inguinal) lymph nodes and stained with anti-mouse CD11c, anti-mouse CD80, and anti-mouse MHCII antibodies.

### Cell culture

MC38 cells (mouse colon cancer cells) were purchased from ATCC (American Type Culture Collection, Virginia, USA) and were cultured in DMEM (Welgene), supplemented with 2 mM glutamine and 10% fetal bovine serum (FBS; Atlas Biologicals) at 37 °C and 5% CO_2_. Bone marrow-derived macrophages (BMDMs) were isolated from mouse femurs and tibias and differentiated for 6 days on petri dishes in BMDM medium [RPMI 1640, 10% FBS, recombinant M-CSF (30 ng/mL), 1 mM sodium pyruvate, 2 mM L-glutamine, penicillin/streptomycin (100 μg/mL)]. Adherent BMDMs were detached on day 6, plated 6-well culture plates (10^6^ cells/well) (Kim et al. 2017; Ahn et al. 2021). For M1/M2 polarization experiments, BMDMs were stimulated with LPS (100 ng/mL) and IFN-γ (10 ng/mL) for M1, and IL-4 (20 ng/mL) for M2 for 24 hours and analyzed for gene and protein expression.

### Immunohistochemistry

Tissues and tumors were embedded in the OCT compound, and 8 µm sections were prepared and stained with Hematoxylin and eosin Y solution (H&E) for histologic evaluation via light microscopy (KPNT, Korea). Sections were photomicrographed with a digital camera mounted on a light microscope (Olympus BX51, Japan), digitized, and analyzed. Analysis was performed on 10 fields of a section at 20x magnification.

### RNA isolation and RT-qPCR

Total RNA was isolated from cells or tissues using the TRI Reagent (Molecular Research Center) according to the manufacturer’s protocol. First-strand complementary DNA was synthesized from 1 to 3 μg of total RNA using reverse transcriptase (Enzynomics). RT-qPCR analyses were performed using SYBR Green Master Mix (Toyobo) and the StepOnePlus Real-Time PCR System (Applied Biosystems) (H. Lee et al. 2020). Expression levels of genes of interest were normalized to those of a housekeeping gene and are presented as fold changes over baseline using the ΔΔCt method. The primers included mouse *Il-6* (sense 5’-ATGAACAACGATGATGCACTT-3’, antisense 5’-TATCCAGTTTGGTAGCATCCAT-3’); mouse *iNos* (sense 5’-AATCTTGGAGCGAGTTGTGG-3’, antisense 5’-CAGGAAGTAGGTGAGGGCTTG-3’); mouse *Arg-1* (sense 5'-ACCTGGCCTTTGTTGATGTC-3’, antisense 5'-AGAGATGCTTCCAACTGCCA-3’); mouse *Vegf-α* (sense 5'-ACAGCACAGCAGATGTGAAT-3’, antisense 5'-ACAGTGAACGCTCCAGGATT-3’).; mouse *Vegf-β* (sense 5'-AAGCCAGACAGGGTTGCCAT-3’, antisense 5'-TGGATGATGTCAGCTGGGGAG-3’).; mouse *Vegfr-1* (sense 5'-ACCGAATGCCACCTCCATG-3’, antisense 5'-AGGCCTTGGGTTTGCTGTC-3’); mouse *Vegfr-2* (sense 5'-GTCTATGCCATTCCTCCCCC-3’, antisense 5'-GAGACAGCTTGGCTGGGCT-3’).

### Statistical analysis

Differences between averages were analyzed using a two-tailed Student’s *t-test*. Data are expressed as means ± SEM.

## Results

### MC38 tumor growth was promoted in IP6K1 KO mice

**Figure 1.**
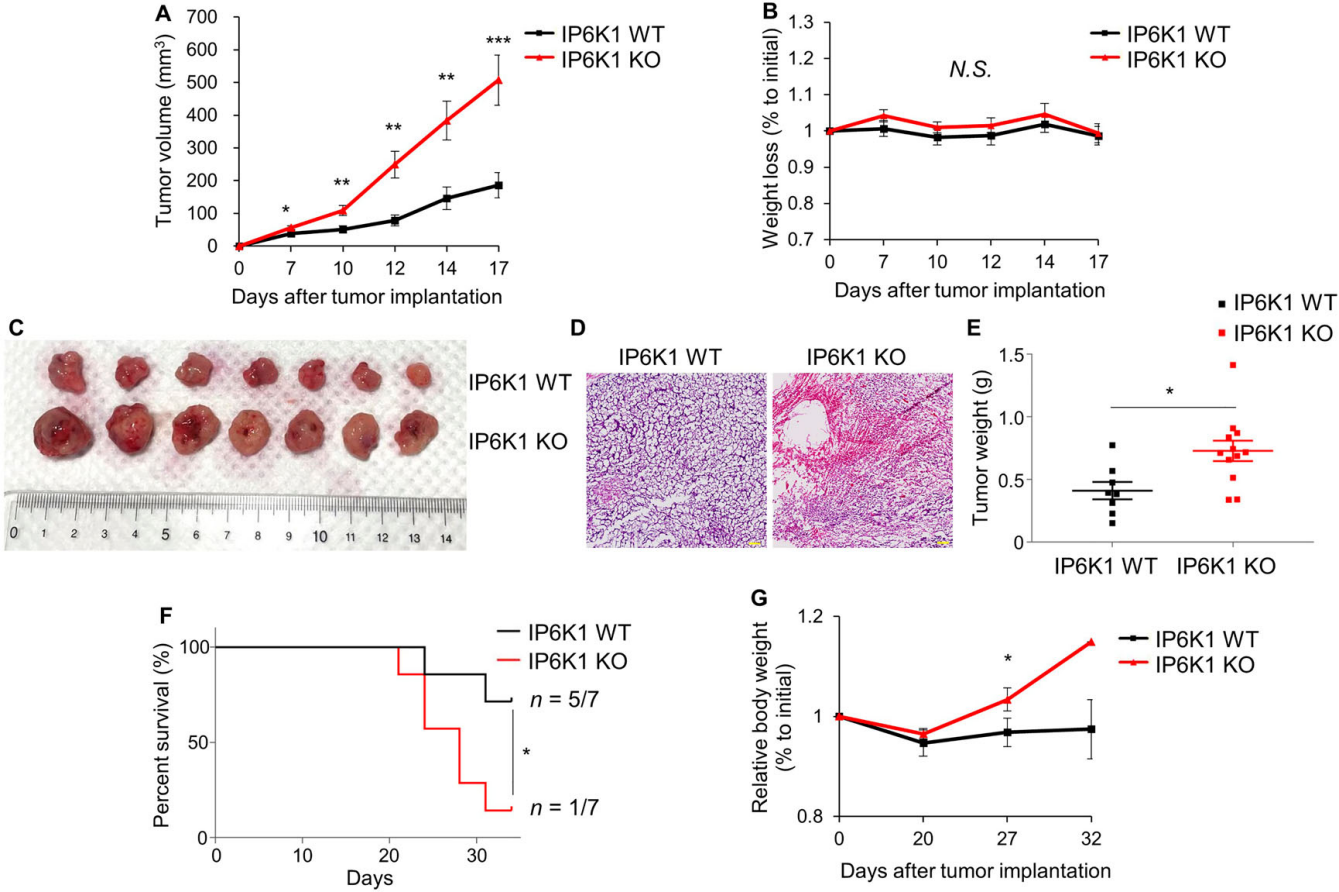
Whole body deletion of *ip6k1* gene aggravates tumor growth. (A) Tumor growth curves of IP6K1 WT (n = 7) and IP6K1 KO (n = 9) mice inoculated MC38 mouse colon cancer cells (2.5×10^5^ cells/side). (B) Relative body weight changes of MC38 tumor-bearing mice during the in vivo experiments. (C) Representative images of tumor tissues at 17 days following inoculation of MC38 cells. (D) Hematoxylin and eosin (H&E) staining of tumor tissues at the end day of experiments. Representative images are shown. Scale bars, 100 μm. (E) Tumor weight assessment at the end day of in vivo experiment. (F) Survival rate of IP6K1 WT (n = 7) and IP6K1 KO (n = 7) mice bearing the MC38 cells (5.0×10^5^ cells/side). The Cumulative proportion surviving was estimated by the Gehan-Breslow-Wilcoxon test. The differences between the groups were statistically significant. (G) Relative body weight changes while measuring survival rate. There is no error bar on day 32 in the IP6K1 KO group because only one IP6K1 KO mouse survived. Data are expressed as means ± SEM (*P < 0.05; **P < 0.01; ***P < 0.001, Student’s *t-*test).

To determine whether host IP6K1 alters tumor growth in C57BL/6 mice, WT and IP6K1 KO mice were injected with syngeneic MC38 mouse colon carcinoma cells and observed for primary tumor growth and survival. The tumors between the two groups started showing significant differences on day 7 post-implantation (Figure 1A). IP6K1 KO mice had markedly accelerated tumor growth by day 17, without notable changes in body weight (Figures 1B−E). Accordingly, we found that mean survival time was significantly decreased in tumor-bearing IP6K1 KO animals, compared to WT mice (Figure 1F). Bodyweight became significantly increased in IP6K1 KO mice after day 27 post-tumor inoculation (Figure 1G), which reflects the higher tumor mass in IP6K1 KO mice compared to control. Collectively, these results indicated the lack of IP6K1 in mice accelerated tumor growth and shortened host survival, suggesting the *in vivo* role of host IP6K1 as a tumor suppressor.

To explore the biology underlying the increased tumor burden in IP6K1 KO mice, we examined indicators of proliferation and angiogenesis. No differences were observed in protein levels of proliferating cell nuclear antigen (PCNA) and retinoblastoma protein (Rb) in tumor tissue from IP6K1 KO compared with WT mice (Supplementary Figure S1A). Both Akt and Erk phosphorylation levels were also unchanged in tumor tissues from IP6K1 KO mice (Supplementary Figure S1B). Similarly, there was no change in the mRNA levels for angiogenic factors such as vascular endothelial growth factor (VEGF) and its receptor (VEGFR) between tumors grown in IP6K1 KO or WT hosts (Supplementary Figure S1C). These results collectively suggest no major role of host IP6K1 in supporting tumor-intrinsic growth properties.

### IP6K1 KO mice show tumors with increased infiltration of immune cells with reduced M1 macrophages

The tumor microenvironment consists of multiple cell types, including various immune cells (Kenny et al. 2007; Hanahan and Coussens 2012). To examine if the deficiency of host IP6K1 in the tumor microenvironment leads to changes in lymphocytes or inflammatory cells during tumor progression, we harvested tumor-bearing tissues from IP6K1 KO and WT mice 2.5 weeks after the injection of MC38 cells and compared the type and quantity of infiltrating immune cells. Flow cytometry analysis revealed increased total viable CD45.2^+^ immune cells in tumors grown from IP6K1 KO mice (Supplementary Figure S2A), suggesting more tumor-infiltrating immune cells in the IP6K1 KO tumor microenvironment.

First, we measured CD11b^+^Gr1^+^ myeloid cells and found no apparent changes in tumor tissues grown from both WT and IP6K1 KO mice (Supplementary Figure S2B). However, tumors from IP6K1 KO mice contained increased levels of CD11b^+^Gr1 ^+ ^IL10^+^ cells compared to control tissues (Supplementary Figure S2C). These results suggest that host IP6K1 deletion leads to more development of immunosuppressive tumor-associated neutrophils/monocytes, presumably establishing a highly immune-suppressive environment to promote tumor growth.

**Figure 2.**
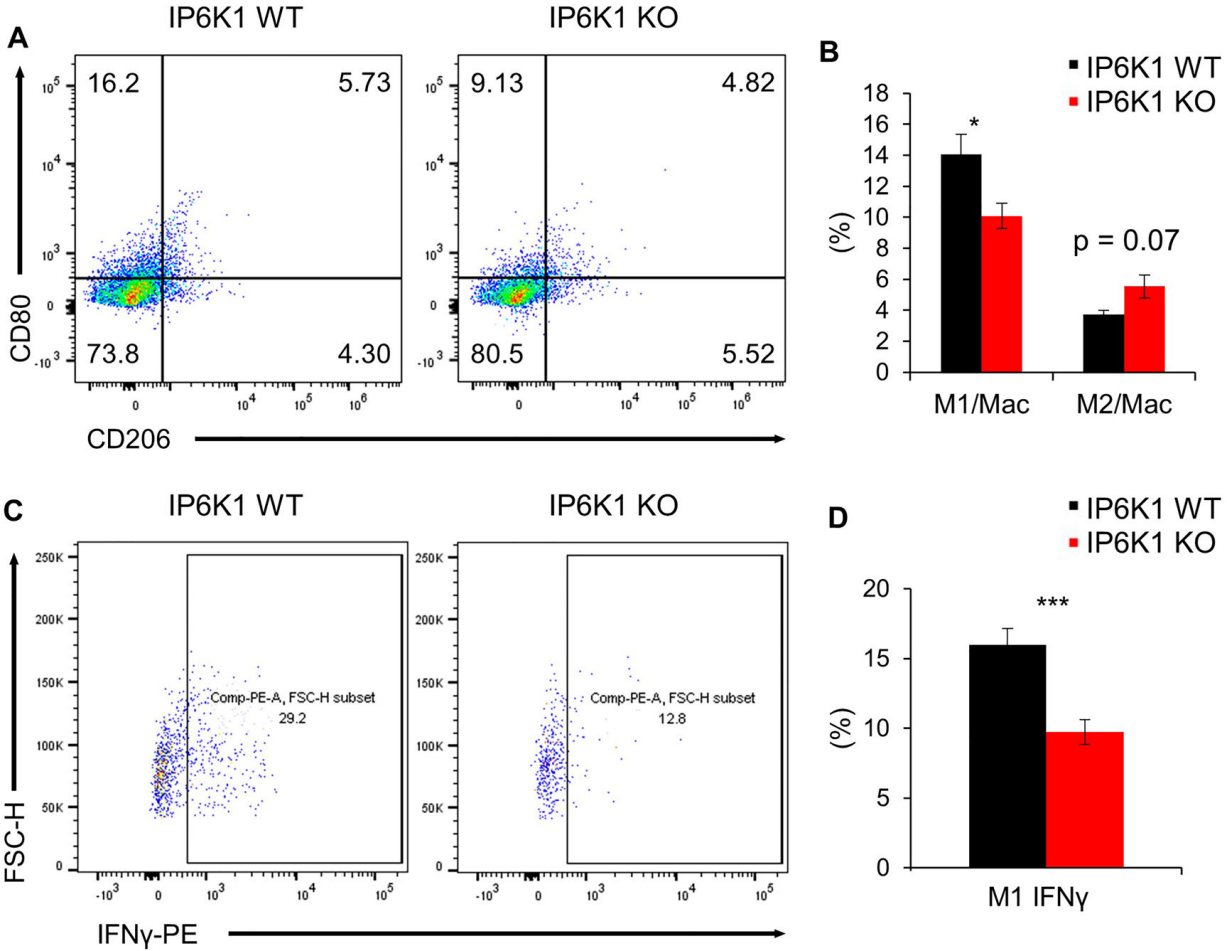
Global depletion of *ip6k1* gene alters tumor-associated macrophage signatures. Flow cytometry analysis of MC38 tumor-associated macrophages (Live cells were pre-gated with CD45.2). (A) Macrophage subpopulations were divided into M1(CD11b^+^F4/80 ^+ ^CD80^+^) and M2 (CD11b^+^F4/80 ^+ ^CD206^+^). The average proportion of M1 to total macrophage was 14.1% (IP6K1 WT, n = 6) and 10.1% (IP6K1 KO, n = 6). The average ratio of M2 to total macrophage was 3.7% (IP6K1 WT, n = 6) and 5.5% (IP6K1 KO, n = 6). (B) The average percentile of each population was presented as a bar graph. (C) Interferon gamma positive M1 macrophage was determined (WT: 16.0%, KO 9.7%). (D) The average proportion of each population was presented as a bar graph. Data are expressed as means ± SEM (*P < 0.05; **P < 0.01; ***P < 0.001, Student’s *t*-test).

To further dissect changes in leukocyte populations, we next analyzed tumor-associated macrophages (TAMs) and identified significantly decreased CD80^+^ M1-polarized macrophages from tumor tissues in IP6K1 KO mice (Figure 2A). A marked reduction of the CD80^+^ IFN-γ M1 macrophages was further noted in the same tumor tissues from IP6K1 KO mice (Figure 2B). These findings suggest that IP6K1 deficiency leads to more anti-tumorigenic, pro-inflammatory M1 polarization in tumor tissues. Since M1 macrophages can suppress tumor growth by directly killing tumor cells or indirectly controlling Th1 cell activities, diminished M1 TAMs found in tumors from IP6K1 KO conditions could contribute to the accelerated tumor growth. We further attempted *in vitro* macrophage polarization by using primary culture of bone marrow-derived macrophages (BMDMs). Levels of M1 markers (e.g. Il-6, iNOS) were rather increased in IP6K1 KO BMDMs, compared to control cells (Supplementary Figure S3A). Furthermore, major activities of Akt and other inflammation signaling events were similar between *in vitro* polarized IP6K1 KO and WT BMDMs (Supplementary Figure S3B), which further indicates that IP6K1 deletion may elicit its impact on TAMs throughout the *in vivo* tumor microenvironment but not *in vitro* culture conditions.

### IP6K1 KO mice show tumors with decreased infiltration of dendritic cells

**Figure 3.**
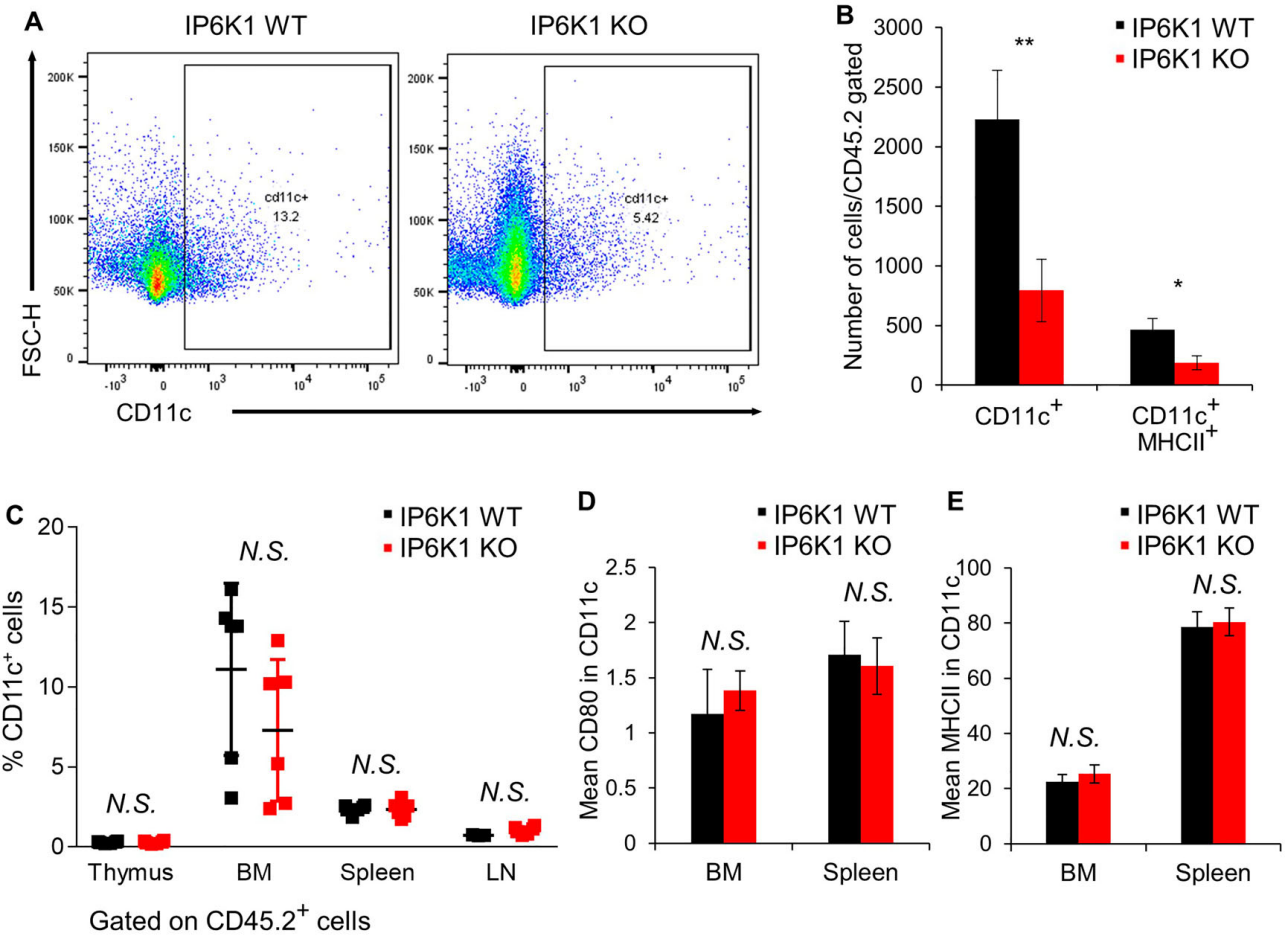
Global depletion of *ip6k1* gene reduces dendritic cell population adjacent tumor microenvironment. Flow cytometry analysis of MC38 tumor infiltrated dendritic cells (DCs, Live cells were pre-gated with CD45.2^+^, CD11b^+^, F4/80^-^). (A) The average DC population was remarkably reduced in IP6K1 KO tumors (65%). MHCII in CD11c^+^ DCs were also reduced in IP6K1 KO tumors (60%). (B) Quantification of CD11c^+^ cells and CD11c^+^MHCII^high^ cells were shown. (C-E) Flow cytometry analysis of dendritic cells within thymus, bone marrow (BM), spleen, or lymph nodes (LN). Live cells were pre-gated with CD45.2^+^, CD11c^+^. (C) percentages of CD11c^+^ cells in different lymphoid organs. (D-E) activation marker expression in CD11c^+^ cells from WT and IP6K1 mice (N ≥ 3). Data are expressed as means ± SEM (*P < 0.05; **P < 0.01; ***P < 0.001, Student’s *t-*test).

In addition to M1 TAMs, dendritic cells (DCs) are another major myeloid cell that contributes to anti-tumor immunity associated with a favorable outcome (Wylie et al. 2019; Wculek et al. 2020). The key role of conventional DCs in anti-tumor immunity depends on their ability to present tumor antigens and to secrete various cytokines to regulate T cell survival and their effector functions (Garris and Luke 2020). The infiltration of total CD11c^+^ and CD11c^+^MHCII^+^ DCs was markedly decreased in tumors formed in IP6K1 KO mice than in the tumors grown in WT control mice (Figures 3A and B).

We further analyzed the effect of IP6K1 on dendritic cell homeostasis. We examined percentages of dendritic cells within the bone marrow (BM) and peripheral lymphoid organs. IP6K1 deletion did not alter the percentages of CD11c^+^ cells within the thymus, BM, spleen, or peripheral lymph nodes (Figure 3C and Supplementary Figure S4). Furthermore, the expression of activation markers such as CD80 and MHCII was comparable to control CD11c^+^ cells (Figures 3D and E). To further examine whether the depletion of IP6K1 affects the dendritic cell maturation *in vitro*, we cultured WT and IP6K1 KO bone marrow dendritic cells (BMDCs) *in vitro* with GM-CSF and IL-4 for 9 days. BMDC maturation induced by lipopolysaccharide led to a robust elevation of CD80 with no difference between WT and IP6K1 KO BMDCs (Supplementary Figure S5), indicating that *in vitro* DC maturation is not affected by the loss of IP6K1. We also investigated whether loss of IP6K1 likewise affects dendritic cell migration. Under the stimulation with CCL5, the number of both WT and IP6K1 KO BMDCs similarly migrated towards the CCL5 (Supplementary Figure S6), suggesting that loss of IP6K1 does not affect *in vitro* migration of dendritic cells.

### IP6K1 KO mice develop tumors with reduced infiltration of CD8 T cells

**Figure 4.**
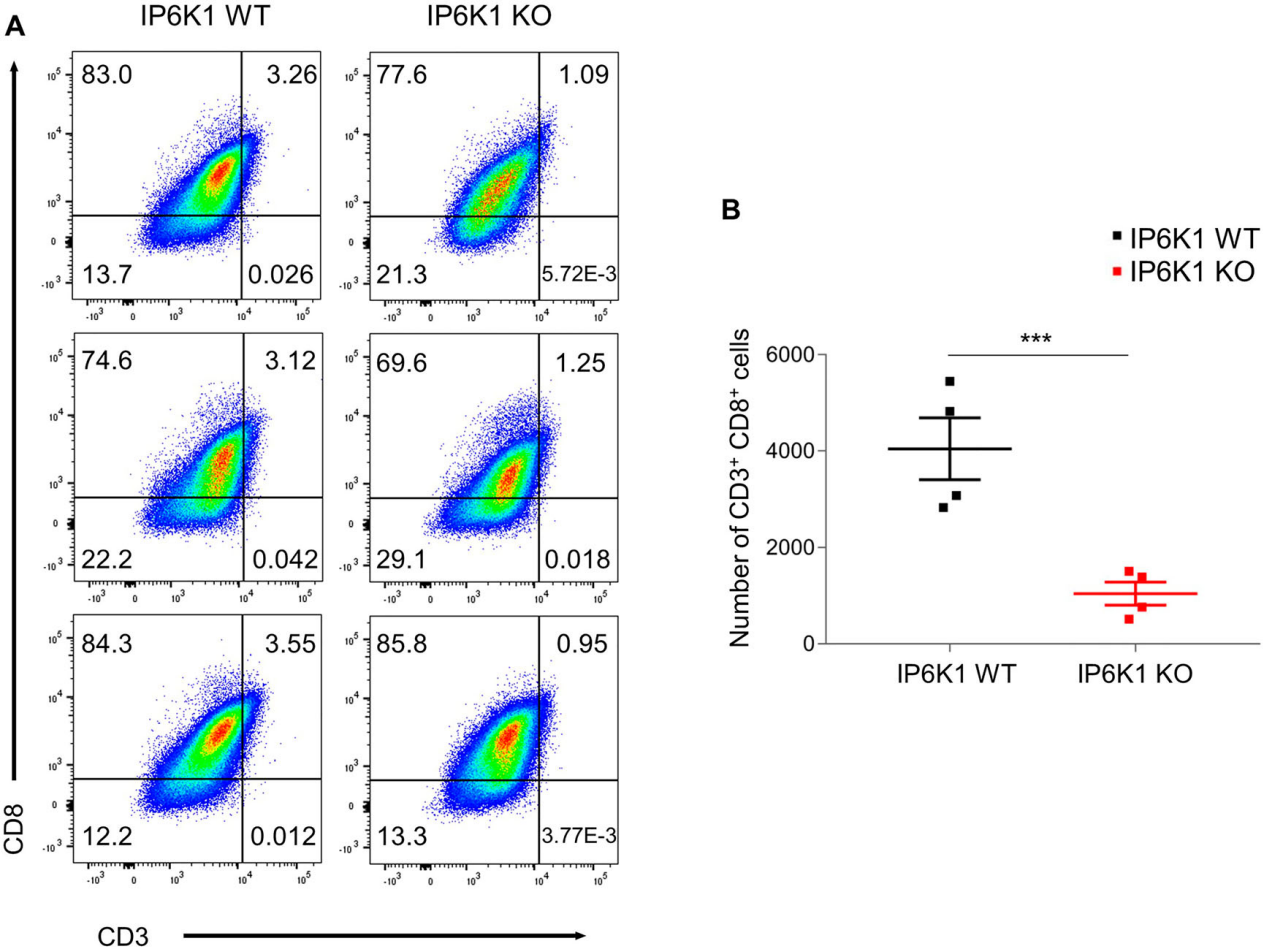
Host IP6K1 KO hinders tumor CD8^+^ T cell infiltration. Flow cytometry analysis of MC38 tumor infiltrated CD3 ^+ ^CD8^+^ T cells (Live cells were pre-gated with CD45.2). (A) The average proportion of CD3 CD8 double positive T cells were 3.3% in IP6K1 WT while 1.09% in IP6K1 KO tumors. (B) Median number of CD3 ^+ ^CD8^+^ cells in IP6K1 WT (3,951 cells / 200,000 total cells gated) and IP6K1 KO (1,075 cells / 200,000 total cells gated) tumors.

Besides myeloid cells, T lymphocytes in the tumor microenvironment play central roles in mediating host anti-tumor immunity (Durgeau et al. 2018). Cross-priming, a process in which DCs activate CD8^+^ T cells by cross-presenting exogenous antigens, is critical for generating anti-tumor CD8^+^ killer T cell immunity (Zamora et al. 2018; Farhood et al. 2019). DCs within the tumor microenvironment are also known to produce chemokines that recruit CD8^+^ effector T cells into tumor tissue. Accordingly, we analyzed T cell populations and observed decreased amounts of CD8^+^ T cells in tumors grown in IP6K1 KO mice, relative to control tumor tissues (Figures 4A and B). Collectively, these findings indicate that host IP6K1 deletion leads to failure of dendritic cell migration into the tumor microenvironment, thereby limiting activation and infiltration of anti-tumor CD8^+^ T cells, subsequently aiding tumor growth in IP6K1 KO mice.

## Discussion

Here, we evaluated the role of host IP6K1 by using a syngeneic MC38 model, a commonly used preclinical model to investigate the complex actions of tumor cell-intrinsic and extrinsic factors in the control of tumorigenesis. We showed that the depletion of host IP6K1 markedly potentiated the growth of MC38 colorectal adenocarcinoma cells. Therefore, we describe the previously unidentified role of host IP6K1 *in vivo* as a tumor suppressor. FACS analyses revealed i) increased CD11b^+^Gr1 ^+ ^IL10^+^ myeloid cells, ii) decreased anti-tumor, M1 TAMs, and iii) markedly reduced infiltration of dendritic cells into the tumor microenvironment in IP6K1 KO mice. Consequently, a significantly reduced amount of CD8^+^ killer T cells was observed in the tumor tissues, when host IP6K1 was deleted. Collectively, these data suggest a major contribution of host IP6K1 to the immune control of cancer cell growth in the tumor microenvironment.

Infiltration by inflammatory and immune cells is a hallmark of malignant tumors that can play a critical role in mounting an antitumor response (Labani-Motlagh et al. 2020; Murciano-Goroff et al. 2020). Most notably, we observed that tumor tissues of MC38 colon carcinoma in IP6K1 KO mice showed very little CD11c^+^ dendritic cell infiltrate. When dendritic cells sense and present tumor antigens, naive antigen-specific CD4^+^ and CD8^+^ T cells are activated, thereby mediating anti-tumor activities (Gardner et al. 2020; Kim et al. 2021). Therefore, reduced detection of total and CD8^+^ killer T cell population in the tumor tissue from IP6K1 KO mice appears the consequence of defective IP6K1 KO dendritic cells. Thus, these findings suggest that an inefficient antitumor response due to the absence of dendritic cell actions might be the key event for the accelerated growth of MC38 cancer cells in IP6K1 KO mice. In addition, increased levels of IL10^+^ myeloid cells (e.g., neutrophils or myeloid-derived suppressor cells), as well as lowered anti-tumor M1 TAMs from tumor tissues in IP6K1 KO mice, appear to be another major contributing factor to avoid immune surveillance. The next challenge is for us to dissect whether host IP6K1 deletion causes those cellular changes in a cell-autonomous manner or not. Conditional deletion of IP6K1 in different immune cells will be critical to fully understand mechanistic details underlying those cellular defects.

Previous studies also proposed IP6K1 and 5-IP_7_ in the control of cellular migration from specific cell types such as neutrophils as well as cancer (Prasad et al. 2011; Jadav et al. 2016; Fu et al. 2017). When BMDCs were stimulated with CCL5, a major neoplastic tissue-derived chemokine, both WT and IP6K1 KO BMDCs were similarly responded, implying that *in vitro* dendritic cell migration potential may be independent of IP6K1. Marked reduction of IP6K1 KO dendritic cells in the tumor microenvironment may reflect complex molecular and cellular actions *in vivo* conditions but not in oversimplified *in vitro* culture settings. Further analyses will be needed to dissect the contribution of IP6K1 to the control of dendritic cell functions in the tumor tissues by using the conditional deletion of IP6K1 in dendritic cells. Examination of whether IP6K1 in T cells could directly influence their effector functions for anti-tumor activities is also required.

Since our approach using syngeneic tumor model demonstrate that targeting IP6K1 from the host promotes tumor growth, we believe that the host IP6K1 as a tumor suppressor may contribute to improving the host immune response against tumor progression. Future studies using various types of cancer cells as well as other IP kinase mouse models will be required to fully elucidate complex cell type-specific functions of host IP6K1 in modulating immune responses among tumor cells and their microenvironment. We further expect that therapeutics that modulate the levels of IP6Ks and their activities will be useful in the management of uncontrolled cancer progression.
